# Klotho is highly expressed in the chief sites of regulated potassium secretion, and it is stimulated by potassium intake

**DOI:** 10.1038/s41598-024-61481-w

**Published:** 2024-05-10

**Authors:** Hyun Jun Jung, Truyen D. Pham, Xiao-Tong Su, Teodora Veronica Grigore, Joost G. Hoenderop, Hannes Olauson, Susan M. Wall, David H. Ellison, Paul A. Welling, Lama Al-Qusairi

**Affiliations:** 1 Department of Nephrology, Johns Hopkins University School of Medicine, Baltimore, MD USA; 2 Department of Physiology, Johns Hopkins University School of Medicine, Baltimore, MD USA; 3 Department of Nephrology, Emory University School of Medicine, Atlanta, GA USA; 4 Division of Nephrology and Hypertension, Department of Medicine, Oregon Health and Science University, Portland, USA; 5 Department of Medical BioSciences, Radboud Research Institute for Medical Innovation, Radboud University Medical Center, Nijmegen, The Netherlands; 6 Division of Renal Medicine, Department of Clinical Science, Intervention and Technology, Karolinska Institutet, Stockholm, Sweden

**Keywords:** Molecular biology, Physiology, Diseases, Endocrinology, Molecular medicine, Nephrology

## Abstract

Klotho regulates many pathways in the aging process, but it remains unclear how it is physiologically regulated. Because Klotho is synthesized, cleaved, and released from the kidney; activates the chief urinary K^+^ secretion channel (ROMK) and stimulates urinary K^+^ secretion, we explored if Klotho protein is regulated by dietary K^+^ and the potassium-regulatory hormone, Aldosterone. Klotho protein along the nephron was evaluated in humans and in wild-type (WT) mice; and in mice lacking components of Aldosterone signaling, including the Aldosterone-Synthase KO (AS-KO) and the Mineralocorticoid-Receptor KO (MR-KO) mice. We found the specific cells of the distal nephron in humans and mice that are chief sites of regulated K^+^ secretion have the highest Klotho protein expression along the nephron. WT mice fed K^+^-rich diets increased Klotho expression in these cells. AS-KO mice exhibit normal Klotho under basal conditions but could not upregulate Klotho in response to high-K^+^ intake in the K^+^-secreting cells. Similarly, MR-KO mice exhibit decreased Klotho protein expression. Together, i) Klotho is highly expressed in the key sites of regulated K^+^ secretion in humans and mice, ii) In mice, K^+^-rich diets increase Klotho expression specifically in the potassium secretory cells of the distal nephron, iii) Aldosterone signaling is required for Klotho response to high K^+^ intake.

## Introduction

Since its discovery in 1997 as an anti-aging protein^1^, αKlotho (termed here as Klotho) has emerged as a player in several health conditions related to aging including cancer, neurodegenerative, cardiovascular and renal diseases^2,3^. Klotho protein, transmembrane and soluble, is expressed in several tissues, but its highest expression is found in the kidney^1,4,5^. Soluble Klotho is cleaved from the transmembrane protein, released to the plasma to function as endocrine and paracrine substance (For review, refer to Kuro-o^6^). Renal Klotho is the main source of plasma Klotho, and it mediates to a large extent the Klotho anti-aging effect^5,7^. Indeed, kidney specific deletion of Klotho recapitulates the Klotho hypomorphic phenotype^7^, while Klotho deletion from the parathyroid, another organ known by its high Klotho expression, does not alter the gross phenotype or survival^8^, highlighting the relevance of renal Klotho in the anti-aging process.

Klotho regulates ion transporters, including the Transient Receptor Potential Vanilloid cation channel (TRPV5)^9^, the sodium and Pi cotransporter (NaPi2a)^10^, and the K^+^-secreting Renal Outer Medullary K^+^ channel (ROMK)^11^. In-vitro studies have revealed Klotho stabilizes ROMK membrane expression and increases ROMK function^11^. The functional relevance of Klotho in K^+^ balance has been shown in rats, as intravenous injection of soluble Klotho increased urinary K^+^ excretion^11^. Moreover, studies have described components of the renal K^+^-secretion machinery are regulated by dietary K^+^^12–17^. This regulation is required to match K^+^ excretion to K^+^ intake and achieve K^+^ balance. Although in-vivo evidence suggests Klotho plays a stimulatory role in K^+^ secretion, it is unknown if Klotho expression is regulated by dietary K^+^.

In recent years, it has become evident that cells of the late distal convoluted tubules (termed as DCT2) and connecting tubules (CNT) are the chief sites of regulated potassium secretion^13–15^. K^+^ secretion in these segments is regulated by Aldosterone (Aldo)-dependent and Aldo-independent mechanisms^18^. Aldosterone is part of the Renin–Angiotensin–Aldosterone System (RAAS), which targets the kidney to regulate renal Na^+^ reabsorption and K^+^- secretion (for review, refer to McDonough and Fenton^19^). High K^+^ intake increases the secretion of the kaliuretic hormone Aldo, which in turn, activates the cellular machinery of K^+^ secretion. Mice lacking components of the Aldo signaling pathway exhibit abnormal K^+^ homeostasis. Indeed, transgenic mice lacking Aldo-synthase (AS-KO), the Mineralocorticoid Receptor (MR), or the Serum and Glucocorticoid-Induced Kinase (SGK1) exhibit reduced K^+^ excretion and hyperkalemia^20–23^. Clinical reports analyzing the effect of RAAS on Klotho indicated RAAS activation decreased Klotho expression in humans, and showed a negative correlation between Klotho and Aldosterone^24,25^, however, cause-to-effect evidence is still missing. It is worth mentioning that an inhibitory effect of Aldo on Klotho expression is incompatible with a stimulatory role of Klotho in K^+^ secretion. The role of Aldo in Klotho regulation requires further investigation.

In this study, we assessed Klotho expression pattern in the renal K^+^-secreting cells in mice and human. We then investigated the regulation of renal Klotho by dietary potassium and analyzed the role of Aldosterone in this regulation.

## Results

### High expression levels of Klotho in mouse DCT2/CNT

**Figure 1:**
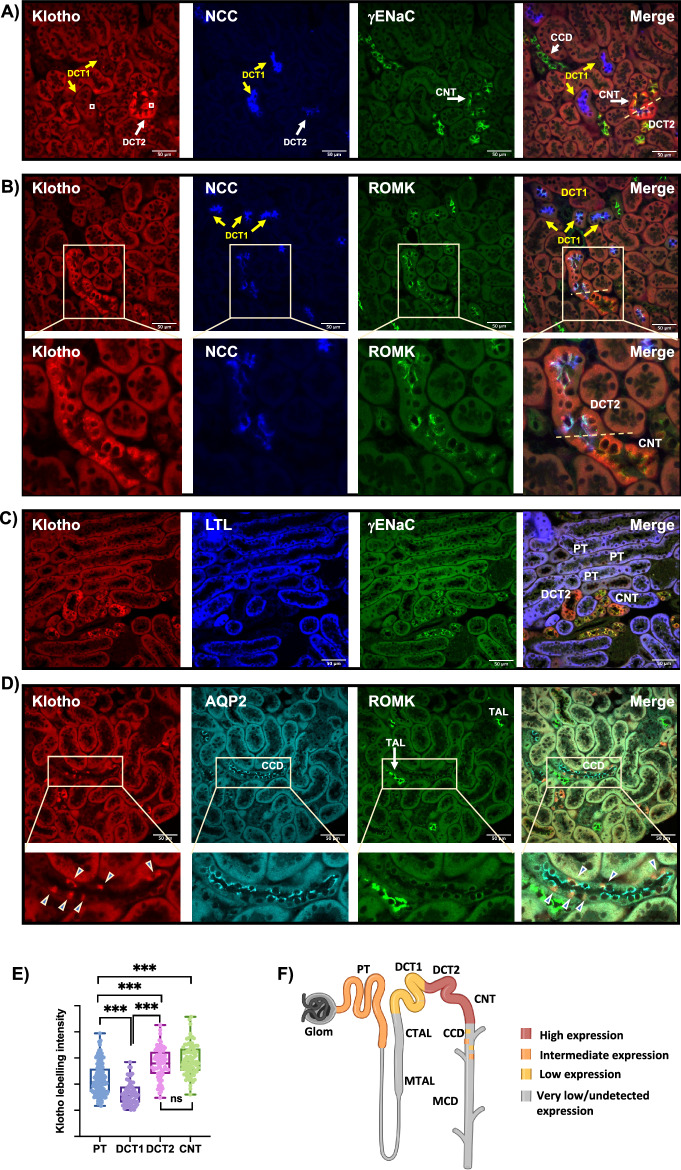
The profile of Klotho expression along the nephron in murine kidneys under basal conditions. (**A**–**D**) Confocal Immuno-fluorescence analysis (× 40) of Klotho in murine kidney showing Klotho localization with segment specific markers. DCT1 and DCT2 were identified by high and low NCC expression respectively (**A**, **B**); CNT were identified as negative for NCC and positive for γENaC and/or ROMK (**A**, **B**); PT were labeled by LTL (**C**); TAL was identified by high ROMK expression (D), CCD was identified by AQP2 labeling (**D**), note some but not all principal cells are labeled by Klotho. No Klotho expression was detected in the glomerulus or TAL. (**E**) Quantification of Klotho labelling intensity in tubular cells from the nephron segments expressing Klotho, data from at least 70 cells/segment from 5 mice were included, white squares in the lefts panel in A represent typical quantified surfaces. One-Way ANOVA was used to assess significance. ***: p < 0.001; ns: nonsignificant. (**F**) Schematic representation of the above data, note the highest Klotho expression along the nephron was detected in the DCT2 and CNT, followed by PT, and to less extent the DCT1, subpopulation of the principal cells (PC) express Klotho.

Nephron tubule microdissection and imaging analysis of mouse kidneys have shown that Klotho exhibits higher RNA and protein expression in the distal than proximal tubules (PT)^10,26^. Here, we investigated if Klotho localizes to specific cells in the Aldo-Sensitive Distal Nephron (ASDN)^13,14^, consisting of DCT2/CNT/CD, where the K^+^ secretion machinery components including ROMK and the epithelial Na^+^ channel (ENaC) reside. We found mouse Klotho is highly expressed in the DCT2 (co-labeled by ROMK, γENaC, and low NCC level), and the CNT (co-labeled by ROMK and γENaC, but negative for NCC) (Fig. 1A, B). We found that, the principal cell-like but not the intercalated cell-like of the CNT express Klotho (Fig. 1A, B). Klotho is differently expressed in the two cell types of the DCT, being more abundant in the potassium secreting cells of the late DCT (DCT2) than the salt-reabsorbing cells in the early DCT (DCT1) (Fig. 1A, B). Klotho expression in the PT, identified by LTL (Lotus Tetragonolobus Lectin) labeling, was less abundant than in the DCT2/CNT (Fig. 1C), confirming previous findings^10,26,27^. We did not detect Klotho in the glomerulus or the other tubular segments, including the cortical thick ascending limb (TAL), recognized by high ROMK expression (Fig. 1D). Klotho was also detected in a small population of AQP2-positive cortical collecting duct (CCD) cells (Fig. 1D). Quantification of Klotho labeling intensity in tubular cells from at least 70 cells/segment from 5 mice under basal conditions allows the identification of the DCT2/CNT as the segment with the highest expression of Klotho along the nephron, then the PT, and to a lesser extent the DCT1 (Fig. 1E, F).

### High expression levels of Klotho in Human CNT

**Figure 2:**
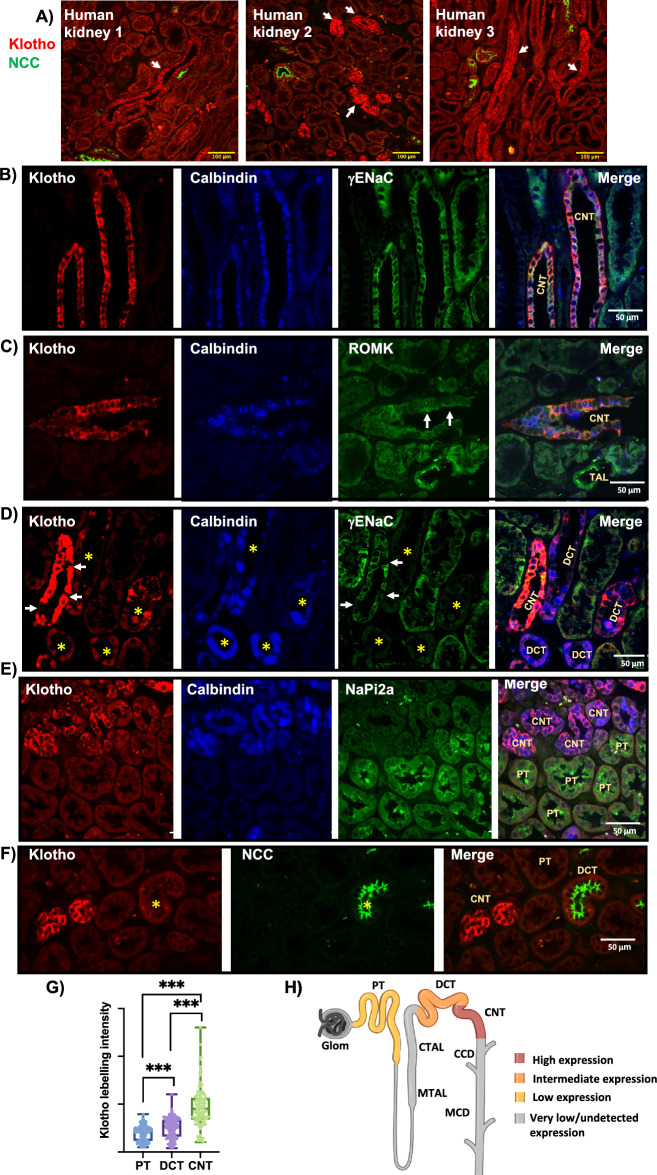
Klotho is highly expressed in the CNT in human kidney. (**A**) Confocal Immuno-fluorescence (IF) imaging (20×) of Klotho in three different human healthy kidneys showing high Klotho intensity in NCC-negative tubules. (**B**, **C**) Confocal IF imaging (40×) of segments with high Klotho intensity, identified as the CNT, as co-labeled by Calbindin and γENaC (**B**); and by Calbindin and ROMK (**C**). Note the basal and apical membrane expression of Klotho in CNT cells, arrows indicate the membrane expression ROMK. (**D**–**F**) Confocal IF imaging with enhanced signal intensity to visualize low Klotho expression in the PT, labeled by NaPi2a, and the DCT labeled by NCC and Calbindin-D_28K_. DCT segments as Calb-Positive, γENaC-negative, or as NCC-positive were indicated by stars. Note Klotho signal in the PT is lower than the CNT and DCT. Note CNT cells, negative for γENaC labeling, are also negative for Klotho indicating Klotho is not expressed in the intercalated cells (arrows in **D**). (**G**) Quantification of Klotho labeling intensity in the PT, DCT and CNT in human kidney. Data from at least 120 cells/segment from 3 donors were included. One-way ANOVA was used to assess significance. ***: p < 0.001. (**H**) Schematic representation of the above data, note the highest Klotho expression along the human nephron was detected in the CNT, then DCT and PT.

To our knowledge, Klotho protein profile along the human nephron has not been investigated. Here, we analyzed the tubular Klotho pattern in the healthy kidney cortex from three male donors. As shown in Fig. 2, Klotho labeling was detected at low intensity in most cortical tubules but was highly abundant in distal tubule segments that stained negative for NCC and colocalized with markers of the CNT including Calbindin-D_28K_, γENaC, and ROMK (Fig. 2A–C). Klotho signal in the CNT was much higher than in the DCT (Fig. 2D, F). Similarly to mice, human Klotho was detected in the principal cell-like but not in the intercalated cell-like of the CNT (Fig. 2D). Klotho expression in the PT, co-labeled by NaPi2a, was very low compared to the CNT and slightly but significantly lower than the DCT (Fig. 2D–F). In contrast to observations in mice, we detected no difference in Klotho expression between DCT1 and DCT2 in human kidneys. Quantification of Klotho signal in at least 120 cells/segment from the 3 human kidneys revealed the CNT is the segment with the highest Klotho expression in the human nephron; Klotho is expressed to a much lesser extent in the DCT and PT (Fig. 2G, H).

### Klotho is regulated by dietary K^+^ intake in the DCT2/CNT

**Figure 3:**
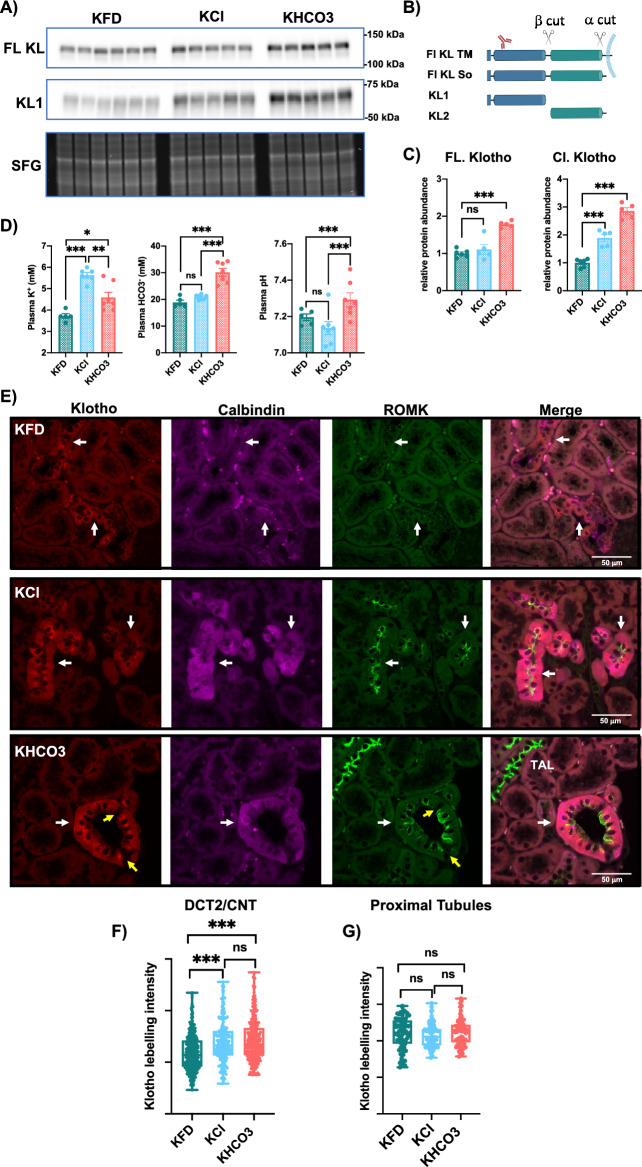
Klotho is regulated by high potassium intake. (**A**) Western blot (WB) analysis of kidney cortex showing Klotho full length and cleaved forms in WT mice fed K^+^-free diet (KFD: 0% K^+^), high KCl (5% K^+^) and high KHCO_3_ (5% K^+^) diets for 4 days. Lower panel shows the image of the stain-free gel (SFG), used as a loading control. (**B**) Schematic representation of Klotho protein showing the full length transmembrane form and the soluble fragments produced by α and β cuts, in addition to the antibody binding site^29^. Note this antibody detects the KL1 fragment, and the full length transmembrane (FL KL TM), and soluble (FL KL So) forms. WB analysis shows KL1 and one band of FL containing both the FL TM and FL So as the difference between them is only 5 kDa^29^. (**C**) Plasma K^+^, HCO_3_, and pH values of the three experimental groups, n = 5–7 mice/group. (**C**, **D**) Quantification of WB in (**A**). (**E**) Confocal IF analysis showing klotho pattern in the early distal nephron (DCT2/CNT, white arrows) as co-labeled by calbindin and ROMK in mice fed KFD, high KCl or KHCO_3_. Note Klotho, Calbindin and ROMK signals are enhanced by high-K^+^ intake and reduced by KFD, indicating they are co-regulated by K^+^. Note ROMK-negative cells do not expressed Klotho confirming observation in Fig. 2D that Klotho is not expressed in the intercalated cells of the CNT (yellow arrows). (**F**) Quantification of Klotho labelling intensity in the DCT2/CNT, data from at least 250 cells from 4 mice/group were included. (**G**) Quantification of Klotho labelling intensity in the PT, data from at least 100 cells from 4 mice/group were included. One-Way ANOVA was used to assess significance. *: P < 0.05, **: P < 0.01, ***: P < 0.001; ns: nonsignificant.

Molecular components of the K^+^ secreting machinery are regulated by K^+^ intake, as low K^+^ diets decrease and high-K^+^ diets increase the abundance and function of channels and transporters, including ROMK, Maxi-K and ENaC^12–15^. We have recently shown that high-K^+^ given as KHCO_3_ enhances K^+^ secretion and the expression of the K^+^-secreting machinery to a greater extent than high KCl^28^. Given the observed expression of Klotho in the K^+^ secreting cells and its previously described role in enhancing K^+^ secretion^11^, we aimed to analyze if Klotho is also regulated by dietary K^+^ intake and its accompanying anion. Here, we used Klotho antibody (KM2076), raised against the extracellular N-terminus, which allow the detection of the full length (FL ~ 130 kDa) and the cleaved KL1 (~ 65 kDa) forms (Fig. 3A, B), consistent with previous reports^29,30^. The detection of the secreted forms in tissue lysates might be due to the fact that Western blotting (WB) is usually performed in non-perfused kidneys, then containing plasma and urine. We analyzed Klotho in cortical preparations from mice fed K^+^-free diet (KFD), high KCl, or high KHCO_3_ diets for 4 days. This dietary treatments result in a higher plasma K^+^ in mice fed high K^+^ diets compared to FKD, and in a higher plasma HCO_3_ and pH in mice fed KHCO3 compared to the other groups (Fig. 3D). As shown in Fig. 3A and C, KL1 is increased by both high K^+^ diets, with KHCO_3_ having a more robust stimulation than KCl. Increased FL Klotho was detected only under KHCO_3_ intake (Fig. 3A, C).

To identify the responsive segment to high K^+^ under the above conditions, we analyzed Klotho expression by confocal imaging in the PT and DCT2/CNT. We found Klotho expression in the DCT2/CNT (Fig. 3E, F), but not in the PT (Fig. 3E, G) was more enhanced under high-K^+^ than low-K^+^ intake. Klotho intensity in the DCT2/CNT cells was increased to a similar extent with high KCl and KHCO_3_ intake (Fig. 3E, F). The fact that higher Klotho expression in KHCO_3_ versus KCl was observed by WB analysis but not by confocal imaging might indicate cellular hyperplasia and/or hypertrophy of Klotho-positive K^+^-secreting cells. Given the short feeding time (4 days), we did not expect major expansion of the DCT2/CNT. We then quantified the cell area of Klotho-positive cells and found this area to be bigger in the high K^+^ fed mice compared to K^+^-deficient mice, but also bigger in the KHCO_3_ compared to KCl group (Fig. S1A, B). The increased cell size of the K^+^-secreting cells is consistent with the expected increase in the workload due to the activation of Na^+^/K^+^ transport under high K^+^ more than low K^+^ intake^13,14^, and under KHCO_3_ more than KCl intake^15^. Indeed, it has been suggested that cells can adapt to a higher workload by increasing their size^31^. Additionally, the greater cell size under KHCO_3_ versus KCl might involve an additional pH-dependent regulation as plasma pH was higher in KHCO_3_ versus KCl groups (Fig. 3D). Indeed, a pH-dependent volume regulation has been previously described in eukaryotic cells^32^. Those adaptations might help increasing the Na^+^-reabsorbing/K^+^-secreting apical surface under K^+^-alkaline-rich diet.

Together, these data indicate that Klotho expression in the DCT2/CNT is stimulated by dietary K^+^ intake, with KHCO_3_ having a more robust effect than KCl.

### Aldosterone is required for Klotho response to the high-K^+^ diet

**Figure 4:**
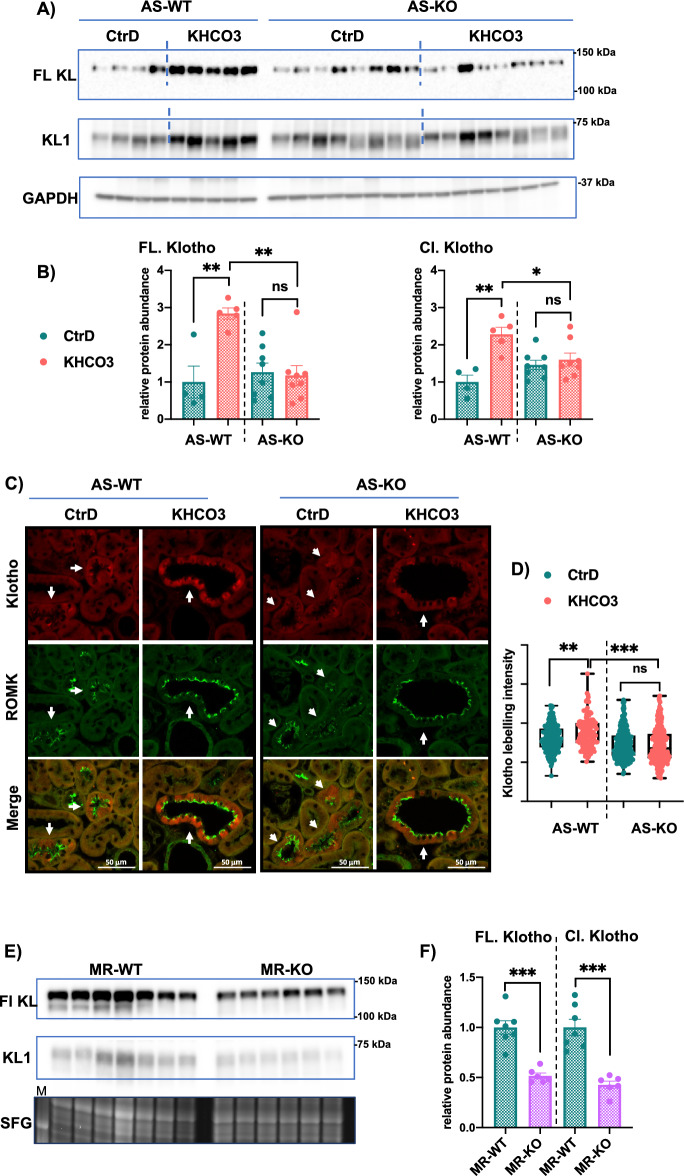
The role of Aldosterone and MR in Klotho regulation. (**A**) WB analysis of kidney cortex showing Klotho full length and cleaved forms in Aldosterone-Synthase KO (AS-KO) mice and WT littermates (AS-WT) fed control (CtrD: 1% K^+^) or high KHCO_3_ (5% K^+^) diets for 4 days. (**B**) Quantification of WB in A. n = 4–5 mice in the WT group, n = 8 mice in the KO group. (**C**) Confocal IF analysis showing Klotho pattern in the DCT2/CNT (white arrows) in the AS-WT and AS-KO mice fed control diet (CtrD: 0.75% K^+^) or high KHCO3 (2% K^+^) for 4 days. Note the blunted Klotho response to increased dietary K^+^ in the DCT2/CNT. (**D**) Quantification of DCT2/CNT Klotho intensity in the 4 groups in (**C**), n = at least 160 cells from 4 mice were analyzed. (**E**) WB analysis of MR-WT and MR-KO mice showing decreased Klotho in the mutant mice. Lower panel shows the image of the stain-free gel (SFG), used as a loading control. M: protein marker. (**F**) Quantification of WB in (**E**), n = 6–7 mice/group. One-way ANOVA was applied in (**B**) and (**D**), unpaired T-test used in (F). *: P < 0.05; **: P < 0.01; ***: P < 0.001. ns: nonsignificant.

Renal potassium secretion is regulated by Aldo-dependent and Aldo-independent mechanisms^20,33,34^. To investigate if Aldo plays a role in Klotho response to high K^+^ intake, we analyzed this response in Aldosterone-synthase (AS) KO mouse model, lacking endogenous Aldosterone^35^. AS-KO mice exhibit normal K^+^ handling under basal condition but became hyperkalemic upon high KHCO_3_ intake (Table S1) confirming previous observations^20,36^. As shown in Fig. 4A and B, the expression of Klotho full-length and KL1 forms were conserved in AS-KO mice under basal conditions. After 4 days of high K^+^ feeding, AS-WT increased Klotho expression, confirming the data in Fig. 3A and C; this response was blunted in AS-KO mice (Fig. 4A, B). Imaging analysis showed that high K^+^ intake in AS-WT mice results in enhanced Klotho expression, specifically in the DCT2/CNT, but this response was absent in the DCT2/CNT of AS-KO mice (Fig. 4C). The quantification of at least 160 cells/condition (40 cells/mouse) showed that KHCO_3_ feeding significantly increases Klotho expression in the K^+^-secreting cells in WT mice but not in AS-KO mice (Fig. 4D). These data indicate that Aldo is required to achieve Klotho response to high K^+^ intake. We further confirmed the regulation of Klotho by Aldo signaling in mice having an inducible deletion of MR in the nephron tubules using the well-characterized Pax8/LC1 system. As shown in Fig. 4E and F, MR-KO mice exhibit reduced Klotho expression compared to the MR-WT mice. These data reveal that high K^+^ intake upregulates Klotho through an Aldosterone-dependent signaling.

## Discussion

Over the past decades, an increasing body of evidence has attributed to potassium-rich diets significant preventive and therapeutic effects. Population-based and clinical studies have reported positive correlation between high K^+^ intake and bone mineral density^37,38^. High K^+^ diet lowers blood pressure in normotensive and hypertensive patients^39–41^, and decreases risk of stroke and cardiovascular diseases^42–44^. Additionally, K^+^ supplementation decreased renal inflammation in chronic kidney disease (CKD)^45^. Interestingly, the physiological effect of high dietary K^+^ parallels in many aspects the biological roles of Klotho. Indeed, Population-based studies have found positive correlation between plasma Klotho level and bone mineral density^46^. Low plasma Klotho is associated with increased arterial stiffness^47^, and with a higher risk of cardiovascular diseases^48–50^. Low plasma Klotho is also correlated with the prevalence of CKD and kidney function decline^51,52^. As mentioned above, the kidney is the main source of circulating Klotho, and the Klotho anti-aging effect is mediated to a large extent by renal Klotho^4,5,7,53^. We have recently described that high KHCO_3_ diet upregulates the machinery of K^+^ secretion and enhances renal K^+^ clearance to a further extent than a high KCl diet, evidenced for a K^+^ balance mechanism that drives adaptation to alkaline/K^+^-rich food^15^. Here, we show Klotho is co-regulated by K^+^ and its accompanying anion in a similar way. Interestingly, increasing KHCO_3_ intake within the physiologic range (from 0.75% K^+^ to 2% K^+^) was sufficient to induce Klotho expression. This observation is of high interest as it helps translating these findings to humans, suggesting that the preventive and therapeutic effects of K^+^/alkaline-rich diet might be at least partially mediated by renal Klotho. Additionally, our data indicate KHCO_3_ exhibits a more robust effect on Klotho expression than KCl, which might be due to a higher plasma HCO_3_ under KHCO_3_ than KCl feeding. These observations are in line with studies in humans showing serum Klotho is positively correlated with serum bicarbonate^54^, and demonstrating that NaHCO_3_ supplementation in CKD patients results in a higher urinary Klotho^55^. The protective effect of NaHCO_3_ on renal Klotho expression has been also described in an oxalate-induced nephropathy mouse model^56^. However, those studies did not address the segment-specific effect of NaHCO_3_ supplementation. Together, this indicates that the more robust effect of KHCO_3_ versus KCl on Klotho expression might include a K^+^-independent/HCO_3_-dependent mechanism.

Here, we show human kidneys express Klotho at a very low intensity in the PT compared to the high Klotho expression in the CNT. Our findings are in agreement with Single-cell RNA sequencing of healthy human kidneys, showing Klotho mRNA was abundant in the distal nephron, but detected at very low levels in the PT^57,58^. We found human Klotho was higher in the CNT than in the DCT, while mouse Klotho showed similar expression in the DCT2 and CNT, but exhibited lower expression in the DCT1. Although differences in the segment-specific pattern of transporters and signaling molecules between small and big mammals are not surprising^59–61^, it is crucial to identify these differences to accurately develop new therapies but also to understand why some murine models do not recapitulate the human diseases. This might be true for Klotho as the function of Klotho in mice differs from human in several aspects. Indeed, Klotho overexpression in mice increases life span, but leads in human to hypophosphatemic rickets and hyperparathyrodisim^62^. Klotho-KO models share common features with patients harboring loss-of-function mutations in the Klotho gene, such as hyperphosphatemia and hypercalcemia^63,64^, However, no Klotho mutations in humans were yet linked to growth retardation, infertility, lung defect, and skin atrophy, as was observed in Klotho KO models^1,65,66^. It is also possible that severe decrease in Klotho levels in big mammals leads to embryonic lethality as has been recently shown in monoallelic knockout fetuses in pigs^67^.

Our data demonstrate that Klotho is most highly expressed in the CNT of human and in the DCT2/CNT of mouse kidneys, which are key sites of regulated K^+^ secretion^18^. Indeed, molecular and electrophysiological studies have found that K^+^ secretion in these segments increase under high K^+^ intake and decrease under low K^+^ intake^13,14^. Our previous work has revealed that the trans-epithelial potassium gradient (TTKG) was increased by both KCl and KHCO_3_ intake compared to the control diet; however, the TTKG was higher under the KHCO_3_ diet than under the KCl diet^15^. Consistent with enhanced expression of the K^+^ secretion machinery in the DCT2/CNT under both KCl and KHCO_3_ intake, with more enhanced effect of KHCO_3_ than KCl^15^. This current study revealed for the first time that Klotho expression in the DCT2/CNT is upregulated by high K^+^ intake with KHCO3 having more robust effect that KCl, consistent with a function of Klotho in stimulating K^+^ secretion, confirming previous findings^11^. The regulation of K^+^ secretion involves Aldo-dependent and Aldo-independent pathways that act concertedly to maximize the kidney capacity to clear K^+^^20,68,69^. Here, we show that AS-KO mice fed control diet exhibit normal plasma K^+^, but they became hyperkalemic under high K^+^ intake, consistent with previous observations^20,36^. Interestingly, AS-KO mice exhibit normal Klotho expression under basal K^+^ intake but they failed to increase Klotho in response to high K^+^ intake, suggesting Klotho might play a role in the Aldosterone-dependent K^+^ secretion. Interestingly, the Klotho hypomorphic model and the kidney-specific Klotho KO are normokalemic under basal K^+^ intake, but they exhibit increased Aldo levels^7,70^, characteristic of Aldo resistance. Indeed, increased plasma Aldo has been observed in several mouse models lacking a chief component of Aldo signaling, such as SGK1-KO^21,71,72^ and MR-KO mice^23^. It is worth mentioning that total and renal-tubular models of SGK1-KO mice are normokalemic under basal K^+^ intake due more likely to a compensatory increase in Aldosterone; but became hyperkalemic upon high-K^+^ challenge^21,22,73,74^. Further investigation is required to understand the role of Klotho in Aldosterone-dependent and -independent K^+^ secretion. Additionally, our experimental design did not allow to conclude about MR role in the K^+^-mediated Klotho regulation. Further investigation is required to assess a role of MR in Klotho regulation in the Aldosterone-sensitive distal nephron.

Here, we show Aldosterone signaling positively regulate renal Klotho expression. These data differ from previous studies suggesting an inhibitory role of Aldo on Klotho. The latter suggestion was based on clinical reports showing high Aldo level is correlated with decreased Klotho^24^; and RAAS inhibition is associated with increased Klotho^75^. Along the same lines, Tang et al. have shown Klotho levels are reduced in WT mice after 3 days of dehydration, concomitantly with increased Aldo level^76^. The authors suggested the reduced Klotho in dehydrated mouse kidneys was mediated by increased Aldo, as they showed Aldo treatment reduced Klotho in HEK cells^76^. This discrepancy might be due to the used antibodies. Our study employed the well-characterized, KO-validated KM2076 antibody generated originally by Kato et al*.*^77^. Indeed, unspecific binding by commercial antibodies has been a major long-standing issue in the field of Klotho biology. Several studies reported the expression of Klotho protein in various tissues, where no expression was detected using other KO-validated antibodies or by gene expression analysis (for review, refer to Olauson^53^). On the other hand, the clinical reports suggesting the inhibitory effect of RAAS on Klotho in CKD patients did not show cause-to-effect evidence. One might suggest that the increased Klotho in CKD patients treated by RAAS inhibitors might be secondary to the improved kidney function.

Together, our data demonstrated that i) the highest expression of Klotho along the nephron is located at the CNT in humans and the DCT2/CNT in mice, where it colocalizes with components of the K^+^ secretion machinery including ROMK and ENaC, ii) Klotho is regulated by dietary K^+^, with more robust stimulation of high K^+^-alkaline than high KCl diet. iii) The regulation of Klotho by dietary K^+^ is mediated by Aldosterone signaling. These findings reveal a new mechanism of renal Klotho regulation and expand our understanding of the benefits of a high K^+^-alkaline diet beyond the specific effect of K^+^ on renal and cardiovascular health.

## Materials and methods

### Animals and treatments

All methods are reported in accordance with ARRIVE (Animal Research Reporting of In vivo Experiments) guidelines, and approved by the Johns Hopkins University Animal Care and Use Committee. All animal methods were carried out in accordance with relevant guidelines and regulations. Male C57BL/6J wild-type (WT) mice were purchased from The Jackson Laboratory. AS-KO and AS-WT littermates were generated by breeding Cyp11b2 ± mice as previously described^35^. Gene deletion in MR Pax8/LC1 mice was induced by doxycycline treatment of adult mice. Doxycycline was administered at 2 mg/ml with 5% sucrose in drinking water for 2 weeks followed by 2 weeks of washout. Food and water were available ad libitum. WT males were randomized to a control diet (1% K^+^; Envigo, cat no: TD.19005), K^+^-free diet (KFD: 0% K^+^; Envigo, cat no: TD.88239), high potassium diet with either potassium chloride (KCl: 5% K^+^; Envigo, cat no: TD.09075) or potassium bicarbonate (KHCO_3_: 5% K^+^; Envigo, cat no: TD.140044) for four days. Experiments on AS-WT and AS-KO mice included two protocols; the first experiment (Fig. 4A, B) included animal fed 1% K^+^ versus 5% K^+^. For ethical concerns, the subsequent experiment was designed using a 3 folds increase in dietary K^+^ within the physiologic range (Fig. 4C, D) (0.75%K^+^ in the control diet, Envigo, cat no: TD.190004 and 2% K^+^ in the KHCO3 diet, Envigo, cat no: TD.230038). Indeed, the 5% K^+^ intake in the absence of Aldosterone results in a severe sickness, more likely due to severe hyperkalemia (7.7 ± 0.4) accompanied by hypovolumia and hyponatremia (plasma Na^+^: 136.4 ± 1)^36^. We found AS-KO mice tolerate better the 2% K^+^ intake as it results in a less severe hyperkalemia (6.1 ± 0.5) with normal plasma Na^+^ (142.2 ±  3) (data on blood analysis are presented in supplementary Table 1). All diets were designed with the assistance of a Teklad-certified dietician and matched for equal caloric intake to the control diet.

### Blood and tissue sampling

For kidney and blood collection, mice were anesthetized by intraperitoneal injection with ketamine-xylazine mix (100 mg/kg ketamine and 10 mg/kg xylazine). Blood was collected from the right common carotid artery, as described before^78^. Blood samples were used immediately for electrolyte analysis (iSTAT-EC8+). Kidneys were removed, and the cortex was separated from the medulla and snap-frozen. Mice were euthanized by exsanguination.

### Human tissues

Healthy kidney samples from 3 male donors (72 y old, 65 y old and 58 y old) were obtained from Maryland Polycystic Kidney Disease Research Resource Consortium (PKD-RRC). Informed consent was obtained from all participants and/or their legal guardians. Human male kidney tissue samples were dissected from nephrectomized human kidneys received from the Baltimore PKD-RRC Clinical Core Center and stored after flash freezing at − 80 °C (reviewed by the University of Maryland Institutional Review Board (UMB IRB) and determined to not be human research, requiring no further IRB review). All experiments were carried out in accordance with Maryland and Federal Human Research Guidelines and Regulations.

### Tissues lysate preparation and western blot analysis

Frozen renal cortical tissues were homogenized in sucrose lysate buffer using a Beadbug device as previously described^78^. Protein separation was performed using gradient (8–16%) Midi-PROTEAN TGX Stain-Free Gels (Bio-Rad; cat no: 5678105). Western blot was carried out as previously described^78^. A list of the antibodies used in this study is presented in Table 1. Luminescence was detected using an Azur 300 imaging system (Azure Biosystems, Dublin, CA). Integrated band intensity was quantified using Fiji software and normalized to GAPDH or to protein bands detected with the Stain-Free Protein Gel.

**Table 1:** Primary antibodies used in this study.

Antibody	Labeled Cell/segment	Host/secondary	WB dilution	IF dilution	Sources
αKlotho		Rat	1/2000	1/100	Cosmobiousa, KAL-KO603 (KM2076)
GAPDH		Mouse	1/10,000		Sigma-Aldrich (G8795)
AQP2	Principal Cells (PCs)	Chicken		1/600	Paul Welling’s Lab^13^
Calb	DCT/CNT	Mouse		1/500	Sigma-Aldrich (C9848)
ROMK	TAL and K^+^ secreting cells in the distal nephron	Rabbit		1/200	Paul Welling’s Lab^13^
γ ENaC	K^+^ secreting cells in the distal nephron	Rabbit		1/200	StressMarques (SPC-405D)
LTL-Biotin	PT	Streptavidin		1/600	Vector Laboratories (SA-5649-1)
NCC	DCT	Rabbit		1/200 (Human)	Sigma (HPA028748)
NCC	DCT	Guinea Pig		1/300 (Mouse)	Paul Welling’s Lab^13^
NaKATPase a-1	Basolateral marker	Mouse		1/200	Millipore (Cl.464 05-369)
NaPi2a	PT marker	Rabbit		1/100	Sigma (HPA077175)
Ezrin	Apical marker	Chicken		1/50	GeneTex (GTX82191)

### Immunofluorescence imaging and quantification

Human and mouse kidney sections (5 μm thick) were prepared from paraffin-embedded tissues, and immunofluorescence staining was performed as previously described^15^. Briefly, sections were deparaffinized and hydrated, epitope retrieval was obtained by 30 min of heating at low pressure in Trilogy solution (No. 920 P-04, Cell Marque). Blocking was performed for 1 h with homemade blocking solution (PBS, 1% BSA, 50 mM glycine, and 0.2% Na-azide). Primary antibodies were applied overnight at 4 °C at the dilutions listed in Table 1, and secondary antibodies were applied 90 min at room temperature. Sections were mounted in mounting medium (H-1000, Vector). Image acquisition was performed using a Zeiss LSM 700 confocal microscope. Images were acquired at a constant gain, contrast, pinhole size, and laser power with a calibrated photodetector. Klotho fluorescence signal and cell area were quantified using Fiji software (Fiji Is Just ImageJ, version: (2.0.0-rc-69/1.52n), https://imagej.net/software/fiji/downloads). To accurately identify cell borders, the basolateral membrane was labeled by NaKATPase and the apical membrane was labeled by Ezrin, only cells with distinguished borders and nuclei were included in the quantification.

### Statistical analysis

Data are presented as means ± SE. Statistical analysis was performed using GraphPad PRISM 8. Unpaired Student’s t-test or two-way ANOVA were used as indicated in the legends. Tukey’s test was used for post-hoc analysis of multiple comparisons. The threshold of significance was P ≤ 0.05.

### Supplementary Information

Supplementary Information.

## Data Availability

The datasets used and/or analyzed during the current study are available from the corresponding author on reasonable request.
