# Supplementary material for: Klotho is highly expressed in the chief sites of regulated potassium secretion, and it is stimulated by potassium intake
Source: Sci Rep. 2024 May 10;14:10740. doi: 10.1038/s41598-024-61481-w (PMC11087591; doi:10.1038/s41598-024-61481-w)
Supplement: Supplementary file 1 — Supplementary Information. [file 41598_2024_61481_MOESM1_ESM.pdf]

| | AS-WT | | AS-KO | |
|---|---|---|---|---|
| | CtrD | KHCO3 | CtrD | KHCO3 |
| pH | 7.3595 (+/-0.051) | 7.3994 (+/- 0.041) | 7.34675 (+/-0.037) | 7.4218 (+/-0.078) |
| $HCO_3^-$ | 19.575 (+/-2.68) | 26.62 (+/- 2.75) * | 19.675 (+/-5.38) | 26.32 (+/-2.18) * |
| $Na^+$ | 146.75 (+/-0.96) | 145.4 (+/-1.95) | 145.25 (+/-1.26) | 142.2 (+/-3.27) |
| $K^+$ | 4.85 (0.72) | 5.52 (0.38) ns | 4.75 (0.24) | 6.1 (+/-0.47) * |
| Hct | 34.5 (+/- 3.1) | 36.8 (+/-1/3) | 38 (+/-2.3) | 39.2 (+/-3.6) |
| Hb | 11.7 (+/-1.1) | 12.5 (0.4) | 12.9 (0.8) | 13.34 (1.2) |

**Table S1:** Blood analysis of AS-WT and AS-KO mice fed control (0.7%$K^+$) or $KHCO_3$ (2%$K^+$) diet for 4 days. Note the WT mice were able to efficiently handle the $K^+$ load while the KO mice became hyperkalemic. Blood was sampled from the Abdominal Aortic Artery and measured using iSTAT CG8+ cartridges.

**Fig S1**

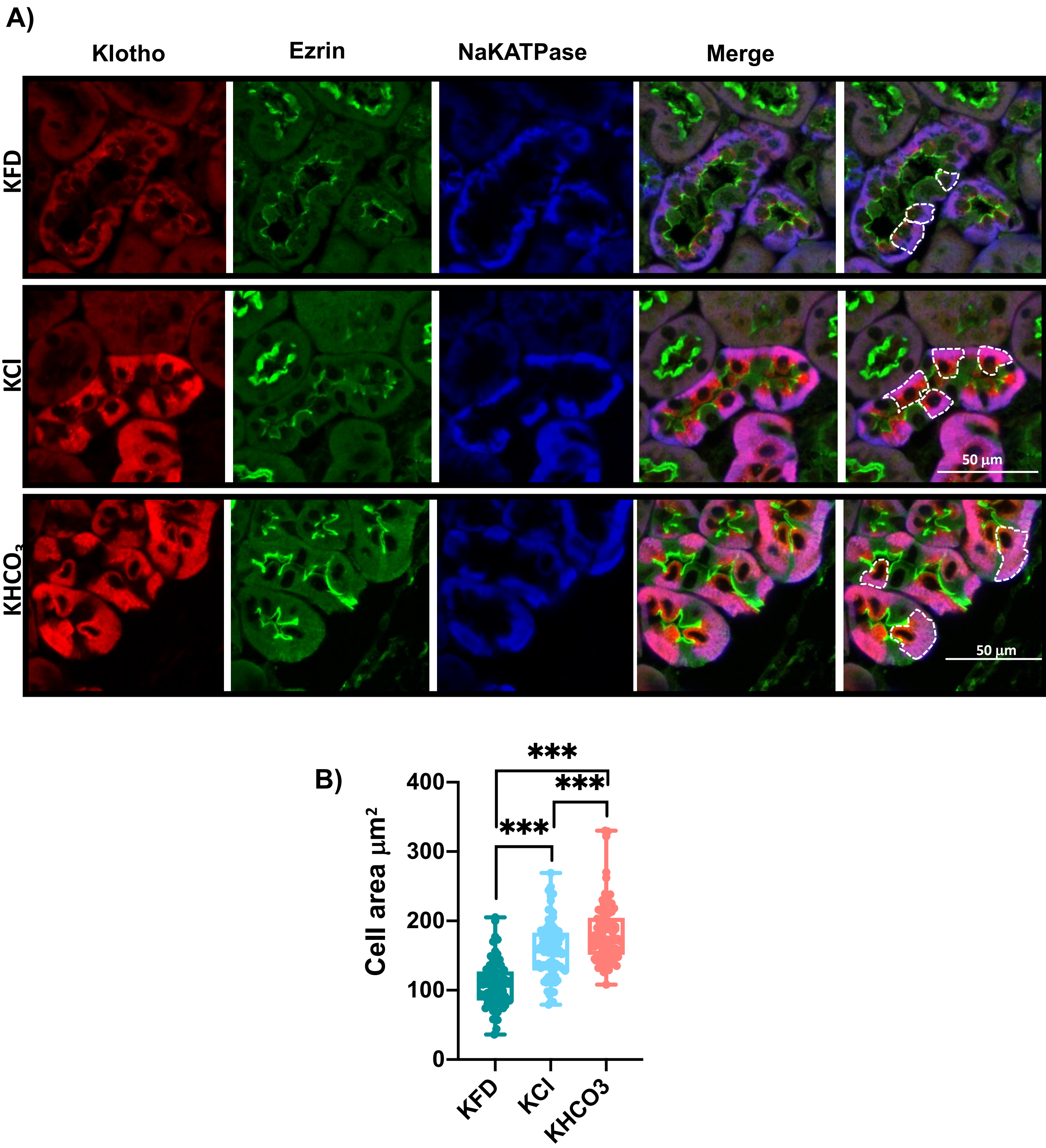


**Figure S1: Increased cell area of Klotho-positive K$^+$-secreting cells by KHCO3 compared to KCl feeding, and by high K$^+$ compared to basal K$^+$ intake.** A) Confocal imaging of kidney cortex in WT mice fed K$^+$-free diet (KFD), or K$^+$-rich diet (5% K$^+$) as KHCO$_3$ or KCl for 4 days, showing Klotho-positive K$^+$-secreting cells co-labeled by NaKATPase (basolateral marker) and Ezrin (plasma membrane marker). B) Quantification of cell area in A, n=at least 120 cells from 4 mice were included. Only cells with distinguish borders and nuclei were analyzed. Doted lines show examples.

**Fig S2**

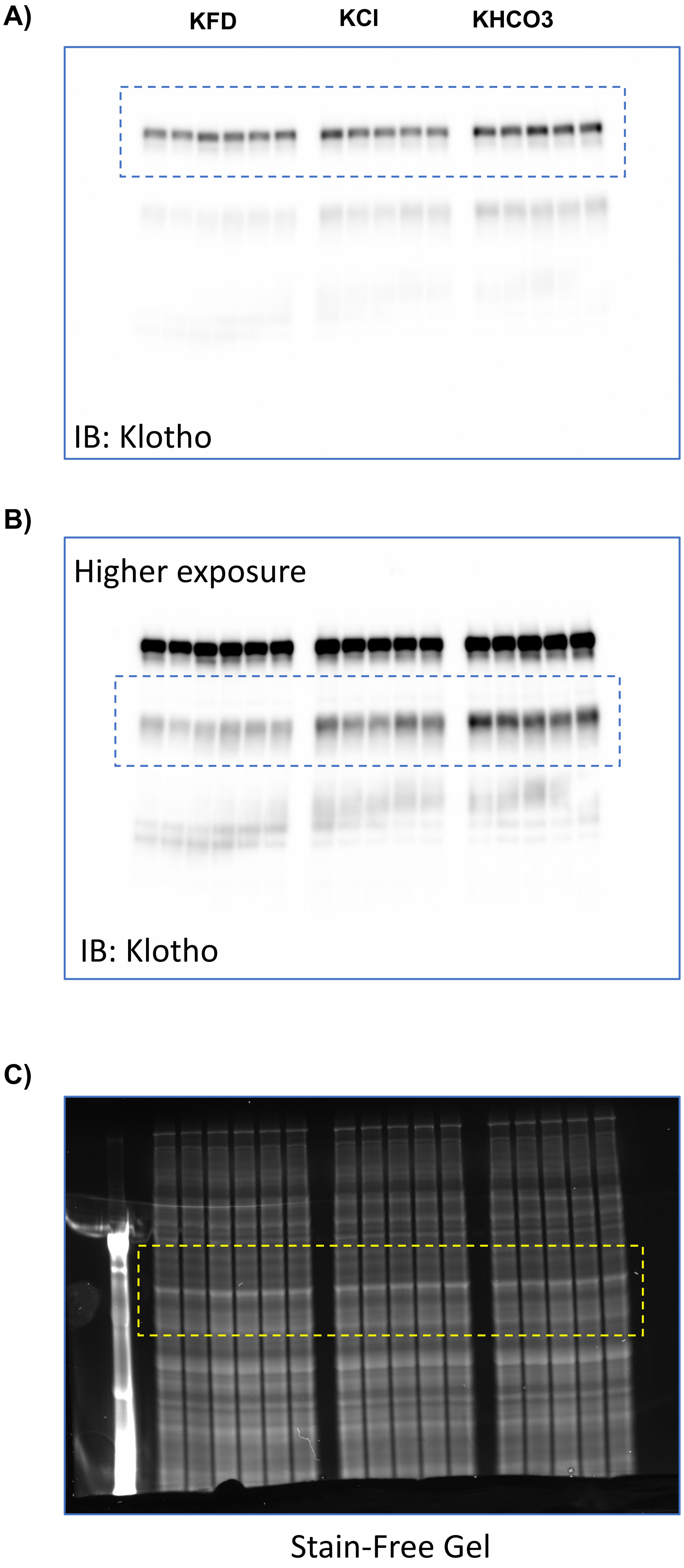


**Figure S2: Original images of Fig 3A.** A,B) Original western blot images of Fig 3A. C) Original images of the related stain-free gel used as a loading control. The doted rectangles indicate the cropped images included in the main figure 3A.

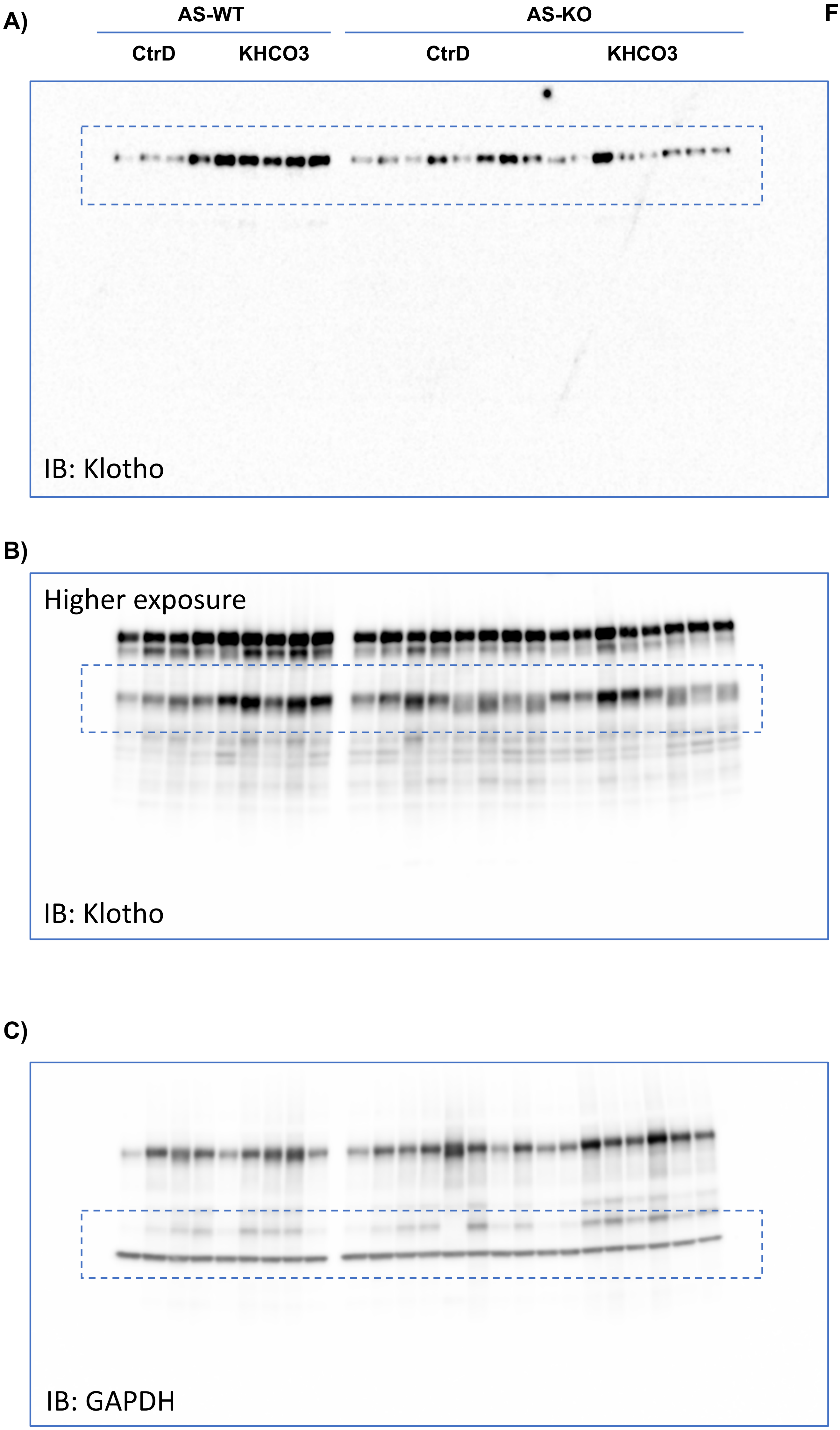


**Figure S3: Original images of Fig 4A.** A,B) Original western blot images of Fig 4A. C) Original images of the related stain-free gel used as a loading control. The doted rectangles indicate the cropped images included in the main figure 4A.

A)

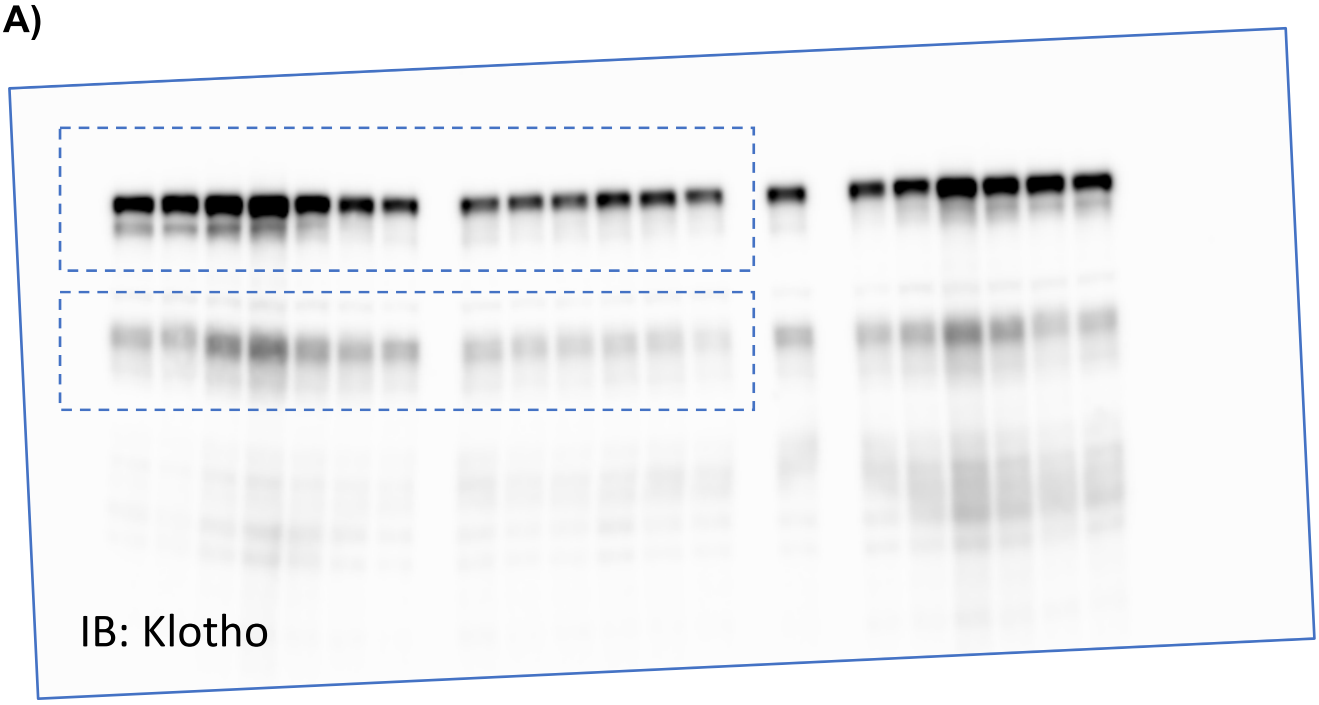


B)

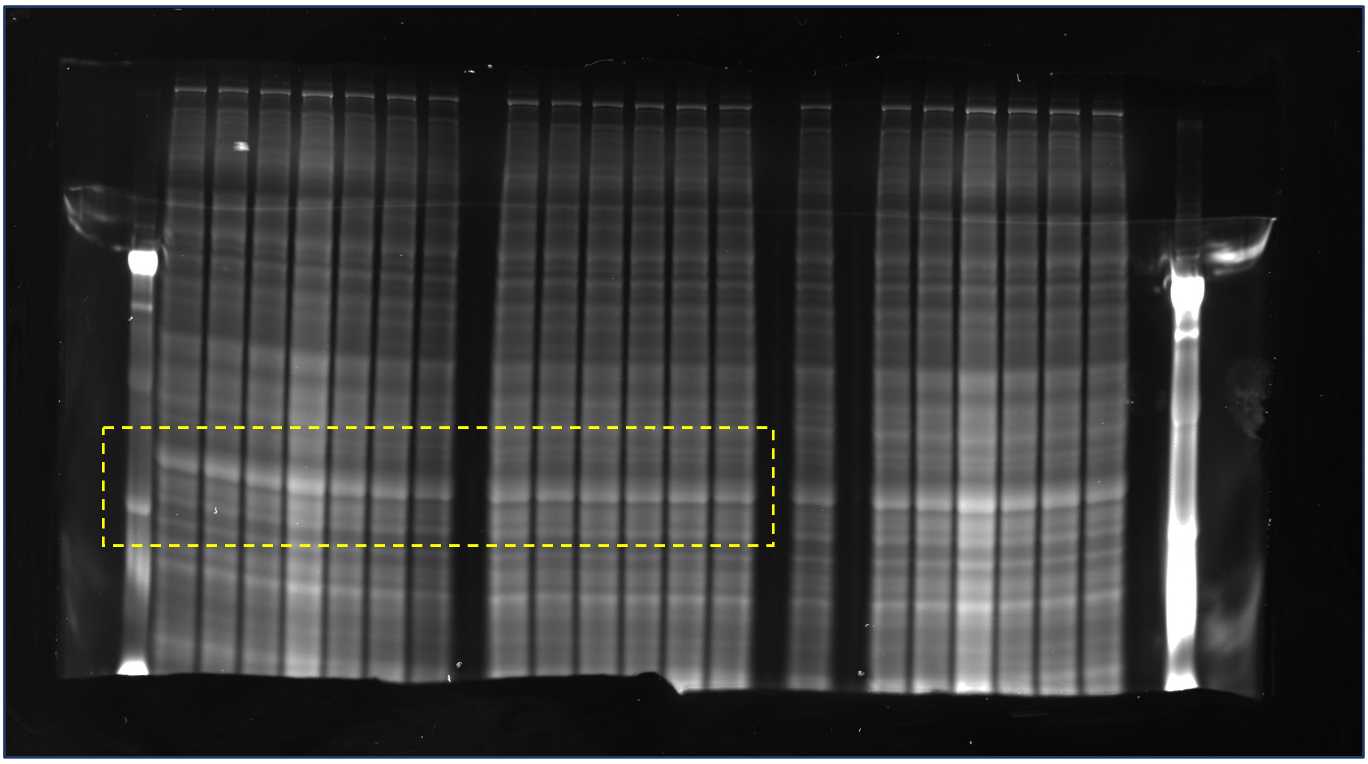

**Figure S4: Original images of Fig 4E.** A,B) Original western blot images of Fig 4E. C) Original images of the related stain-free gel used as a loading control. The doted rectangles indicate the cropped images included in the main figure 4E.
